# Sirolimus combined with glucocorticoids in the treatment of Kasabach-Merritt phenomenon in a neonate: A case report

**DOI:** 10.1097/MD.0000000000037706

**Published:** 2024-04-05

**Authors:** Jun Cheng, Yun Zou, Ronghua Fu, Pingliang Jin, Mengyu Huang, Zhiping Wu, Hanxiang Bai, Xiangqun Huang, Hua Yuan

**Affiliations:** a Department of Plastic Surgery, Jiangxi Provincial Children’s Hospital, Nanchang, China.

**Keywords:** glucocorticoid, Kaposiform hemangioendothelioma, Kasabach-Merritt phenomenon, sirolimus

## Abstract

**Rationale:**

Kaposiform hemangioendothelioma is an aggressive vascular tumor that is often associated with life-threatening coagulopathies and Kasabach-Merritt phenomenon. Pathologic biopsies can provide a good basis for diagnosis and treatment. Therapy with srolimus combined with glucocorticoids may offer patients a favorable prognosis.

**Patient concerns:**

A large purplish-red mass on the knee of a child with extremely progressive thrombocytopenia and refractory coagulation abnormalities. Conventional doses of glucocorticoids alone failed to improve coagulation abnormalities and the child developed large cutaneous petechiae and scalp hematomas.

**Diagnosis:**

Kaposiform hemangioendothelioma combined with Kasabach-Merritt phenomenon.

**Interventions:**

The patient received prednisolone 2.0 mg/kg*d for 4 days. Blood products were transfused to ensure vital signs and to complete the pathologic biopsy. Sirolimus combined with prednisolone was given after clarifying the diagnosis of Kaposiform hemangioendothelioma.

**Outcomes:**

The tumor basically disappeared on examination and the ultrasound showed a subcutaneous hyperechoic mass with normal blood flow.

**Lessons:**

Sirolimus combined with glucocorticoids is effective in controlling Kasabach-Merritt phenomenon and pathologic biopsy is important for definitive diagnosis.

## 1. Introduction

Kaposiform hemangioendothelioma (KHE) is a rare vascular tumor that occurs mainly in infants.^[1,2]^ KHE is locally aggressive and can invade muscles, tendons and bones.^[2]^ Of these, about 70% of KHE will develop into Kasabach-Merritt phenomenon (KMP).^[1]^ KMP is a clinical syndrome characterized by giant vascular tumors with thrombocytopenia and abnormalities of consumptive coagulation. In severe cases, there is a tendency to bleeding and even death due to diffuse intravascular coagulation.^[3]^ Therefore, early diagnosis and effective treatment are particularly important.^[4]^ Recently, a child with knee KHE, complicated by KMP and scalp hematoma received sirolimus combined with methylprednisolone for treating KMP. Progressive platelet drop and consumptive coagulation abnormalities were improved. The tumor was significantly controlled.

## 2. Case presentation

**Figure 1.**
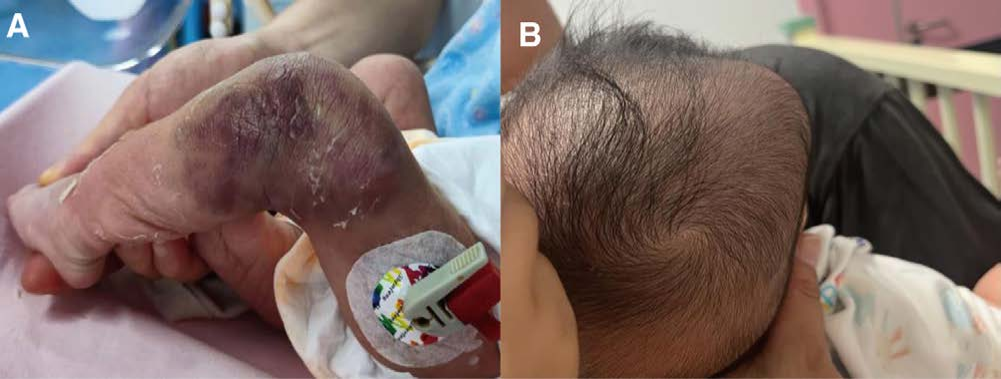
(A) The child had a 10*8 cm hard purple mass on the lateral side of the left knee with uneven color and inability to passively extend the lower extremity. (B) The child’s scalp hematoma increased in size.

A 38 weeks + 1 day full term baby, weight: 2810 g, born with a purplish mass of 10*8 cm on the left knee. Due to progressive platelet decline, he was transferred to our neonatal intensive care unit on the second day of life. His mother’s routine blood tests during pregnancy were normal, and there was no massive bleeding or inability to clot during delivery. No familial inheritance of thrombocytopenia was found. At room temperature without oxygen, the vital signs were stable, the consciousness was clear. A large, purplish, indurated, unclear boundaries mass was present over the left lateral knee that prevents the knee from being passively straightened (Fig. 1). Blood tests revealed: platelet 30 × 10^9^/L, hemoglobin 141 g/L, red blood cell 4.19×/L, fibrinogen 118 mg/dL, D-dimer 13,410 ng/mL. On the same day, 50 mL of fresh frozen plasma were transfused.

**Figure 2.**
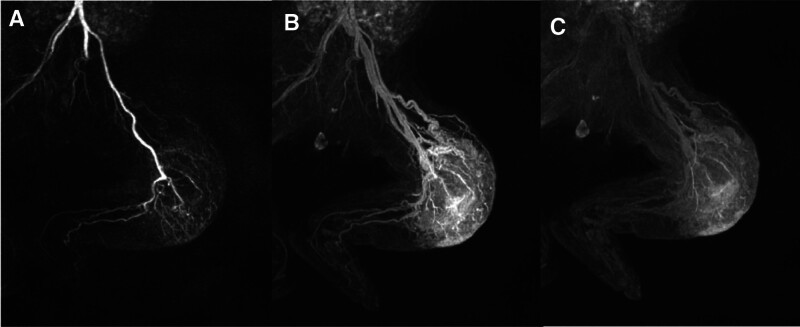
(A) MRI enhancement in the arterial phase shows that the tumor is supplied by a branch of the femoral artery. (B/C) MRI enhancement in the venous and delayed phases show tumor invasion of the muscular layer and reflux through the femoral vein.

Ultrasound of the left leg found an echogenic mass measuring 4.3 × 2.5 × 6.2 cm within the left anterior thigh containing venous waveforms, the boundary was not clear, the blood flow signal was rich. Magnetic resonance imaging (MRI) of the lesion showed a contrast enhanced lesion with multiple vascular channels concerning for a vascular tumor, with no arteriovenous malformations (Fig. 2). MRI revealed isointense signal abnormality on T1-weighted images and hyperintense signal abnormality on T2-weighted images. After combining clinical manifestations and tests and examinations, KMP was considered and eventually managed by the neonatal intensive care unit, plastic surgery, hematology and rheumatology departments.

**Figure 3.**
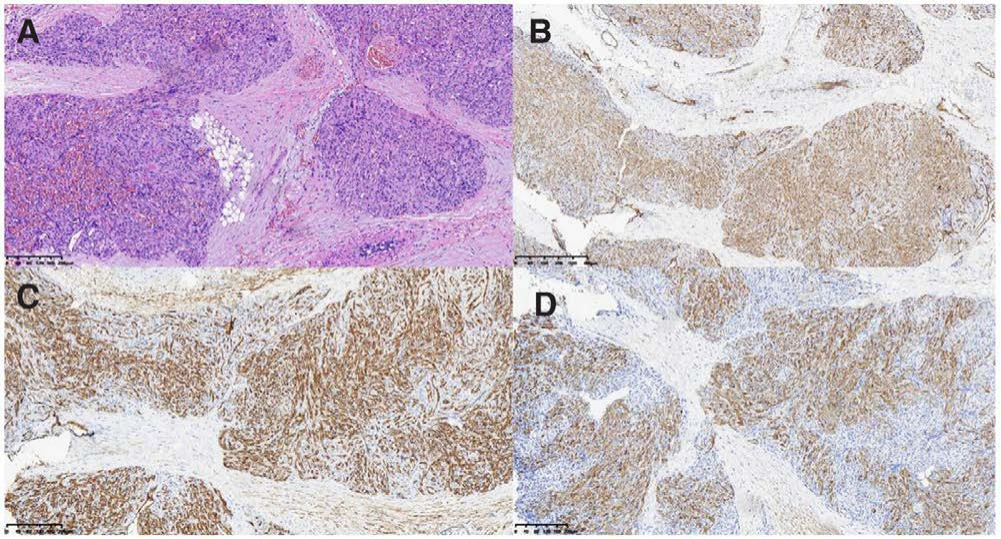
(A) Hematoxylin and eosin staining showed that the tumor was composed of spindle-shaped cells, fissure capillaries. (B/C/D) Immunohistochemical staining showed positivity for CD31,CD34, and D2–40.

The patient received prednisolone 2.0 mg/kg*d for 4 days, during which time there was no significant change in tumor size and no change in platelets, fibrinogen, or D-dimer. On the third day of medication, a hematoma of about 2*3 cm was found in the patient’s head. We were unable to determine whether the thrombocytopenia and coagulation abnormalities were caused by this tumor or whether the child had a hematologic disorder, such as ITP. Then, the child underwent a local biopsy of the tumor under general anesthesia and was given a preoperative platelet transfusion of 60 mL in the hope of reducing intraoperative bleeding. Pathological results showed that the tumor was composed of spindle-shaped cells, fissure capillaries. Immunohistochemical staining showed positivity for CD31, CD34, and D2–40 but negative for Glut-1, all of which confirmed the diagnosis of KHE (Fig. 3). However, the hematoma in the child’s head increased in size and developed large subcutaneous hemorrhagic petechiae on the upper extremities. We need to raise platelets quickly to prevent more dangerous bleeding such as intracranial and intra-abdominal hemorrhage. Hematology department, rheumatology and immunology department was invited to consult.

On day 5, prednisolone was discontinued and methylprednisolone was given 15 mg/kg*24 h and sirolimus 0.1 mg/24 h once daily after the risks and benefits were explained to the child’s parents and consent was obtained. Four days after the therapy, the patient’s fibrinogen and platelet count gradually normalized, and D-dimer levels immediately decreased. Methylprednisolone and gammaglobulin were discontinued, prednisolone was administered orally once daily at 2 mg, and Sirolimus continues to be taken orally once a day at 0.1mg. During this period, we dynamically adjusted the sirolimus dose to maintain the trough serum concentration between 2 ng/mL and 6 ng/mL. The tumor color continued to fade and the tumor continued to shrink. The changes in the test indexes of the children after drug administration are shown in appendices.

**Figure 4.**
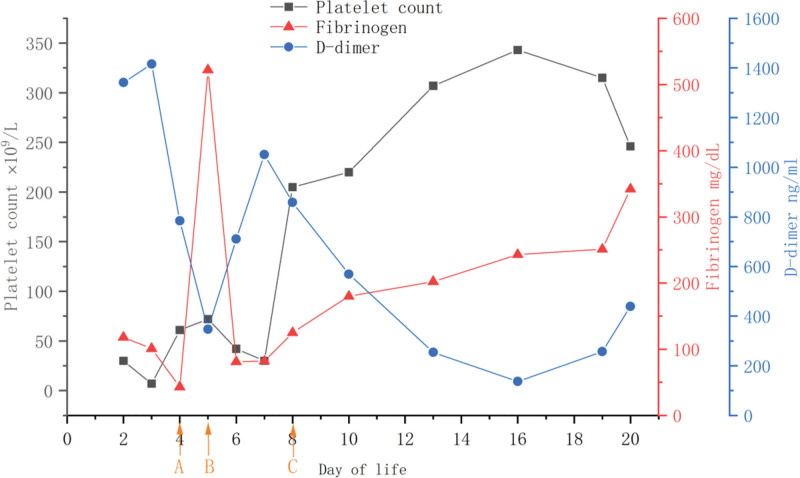
Fluctuations in platelet count, fibrinogen, and D-dimer. (A) The child underwent a local biopsy of the tumor on the fourth day of life. (B) The child receives methylprednisolone and sirolimus. (C) Platelet count, fibrinogen, and D-dimer improved on day 4 of methylprednisolone and sirolimus.

**Figure 5.**
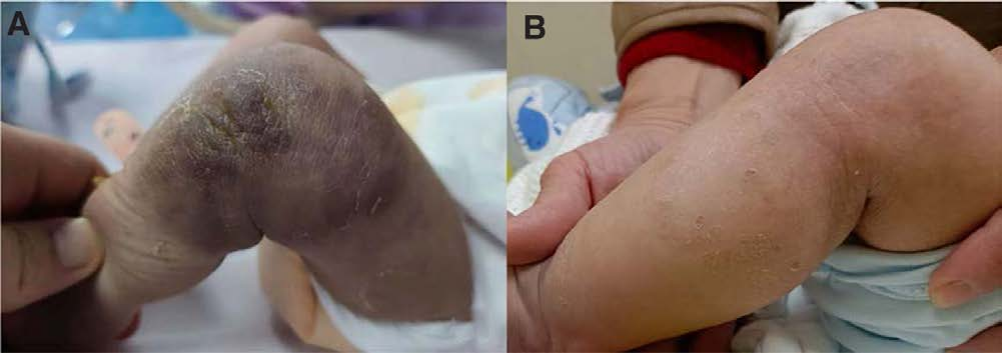
(A) Ten days after the child received triple therapy, the tumor became darker and the skin temperature dropped. (B) Complete tumor regression after 8 months of treatment.

On the 18th day of life, the child underwent a scalp hematoma puncture and compression bandage. Then, the child was discharged on the 22nd day of life. During the entire follow-up period, we gradually reduced the glucocorticoids dose until it was discontinued. Sirolimus dose was dynamically adjusted to maintain the serum concentration of sirolimus at 2 to 6 ng/mL. After 4 months of treatment with oral sirolimus, the tumor continued to shrink, became lighter in color, and was within the normal range of platelets and fibrinogen and D-dimer (Fig. 4). No significant side effects were observed. After 8 months of oral sirolimus, we saw that the tumor basically disappeared on examination and the ultrasound showed a subcutaneous hyperechoic mass with normal blood flow (Fig. 5). As a result, sirolimus was discontinued and the child is still being followed up.

## 3. Discussion and conclusions

Previous reports have shown that the population prevalence of KHE/tufted angioma is 0.91 per 100,000, of which approximately 71% progress to KMP, with infancy being the high incidence age and up to 60% in newborns.^[1]^ KMP develops rapidly, with a mortality rate of 20% to 30%.^[5]^ Therefore, KMP is also considered to be a predictor of poor prognosis. Clinical treatment of KMP is mainly empirical, although a variety of treatments are available, such as surgical resection, pharmacological treatment, and interventional procedures.

Surgical treatment is considered to be a possible radical cure for KHE.^[3]^ Surgical resection can be preferred for lesions located in superficial areas, and intra-tumor artery embolization can be considered for those who cannot be surgically resected; If the general state of health is too poor to tolerate surgery or intervention, drug therapy may be used and surgical treatment can be considered after the tumor shrinks.^[6]^ However, surgical resection in this child was difficult and prone to recurrence due to MRI suggesting extensive infiltration of the anterior femoral muscle group with poorly defined borders and a huge tumor with abundant blood flow. The severe coagulation abnormality, together with the huge surgical trauma, could be fatal for the newborn. Therefore, radical resection was considered impossible for this child.

A large number of clinical studies have reported the effectiveness of systemic glucocorticoid therapy on KHE, with an efficiency rate of 11% to 66%.^[7–9]^ The possible mechanisms by which glucocorticoids can inhibit tumor growth and also counteract extreme platelet depletion are: Glucocorticoids can effectively inhibit vascular endothelial cell proliferation, induce apoptosis, and prevent fibrinolysis and thrombosis, as well as stimulate bone marrow hematopoiesis and platelet release and antagonize platelet antibodies,^[10]^ increase platelet lifespan, and inhibit angiogenesis,^[11]^ all of which can effectively inhibit tumor proliferation and elevate platelets.We administered the recommended dose of glucocorticoids, but there was no elevation of platelets and no significant improvement in coagulation abnormalities. During this period the child developed a large scalp hematoma with multiple petechiae on the skin of the trunk and extremities. Persistent platelet drop and coagulation abnormalities may also lead to intracranial or intra-abdominal hemorrhage or disseminated intravascular coagulation. To prevent potentially life-threatening consequences, we increased the dose of glucocorticoid. However, long-term use of glucocorticoids is prone to associated side effects such as hypertension, obesity, acne, Cushing syndrome, and opportunistic infections.^[12]^ In Recent years, glucocorticoid therapy in combination with other drugs has gradually emerged to reduce the dosage of glucocorticoids or shorten the course of treatment in order to reduce complications. We therefore combined glucocorticoids with sirolimus.

Sirolimus has been used for several years in the treatment of KHE. It has been found that tumor tissues of KHE aberrantly express mTOR protein,^[13]^ a signaling pathway that affects a variety of cell biological features, angiogenesis, cell growth and apoptosis, as well as cellular catabolism and anabolism.^[13,14]^ Sirolimus, an mTOR inhibitor, has achieved better efficacy in the treatment of diseases associated with abnormal PIK3/AKT/mTOR signaling pathway.^[3,8,15]^ Efficacy of sirolimus in the treatment of KHE and slow-flow malformations achieved,^[16–18]^ and the current commonly used dose is 0.8 mg with the trough serum concentration of 10 to 15 ng/mL.^[19]^ However, there are also many studies pointing out that the dose and blood concentration are high, which can easily lead to stomatitis, gastrointestinal reactions, fever, rash, pain and even life-threatening Pneumocystis carinii pneumonia.^[20,21]^ Sirolimus is metabolized by the hepatic enzyme CYP3A4,^[22]^ which is barely expressed in newborns after birth and increases slowly during the first year of life, so the bioavailability and half-life of sirolimus in infants can change.^[23]^ Czechowicz et al suggested a target serum concentration range of 4 to 10 ng/mL for sirolimus.^[24]^ HARBER et al showed that even at very low serum concentration range of 2 to 6 ng/mL, sirolimus remained effective in patients with KHE and KMP without serious adverse effects.^[25]^We attempted to treat KMP with a lower than usual dose of sirolimus in order to reduce the side effects associated with long-term sirolimus use. We dynamically adjusted the serum concentration of sirolimus between 2 to 6 ng/mL, and it seems that maintaining low serum concentration can significantly promote the tumor shrinkage at the current follow-up.

Management of patients with KMP is a very challenging task, especially when combined with other complications. Our experience shows that the development of treatment programs and the use of medications are not static. Glucocorticoids combined with sirolimus have been used to treat KMP for several years and have achieved some efficacy.^[16]^ However, reduced platelets, coagulation abnormalities and side effects of sirolimus seriously threaten the health of the children. Timely blood product infusion can help accomplish some minimally invasive maneuvers such as tissue biopsies and hematoma punctures. Adjust the dose of sirolimus according to the child’s age and weight, for newborns can be appropriate to reduce the blood concentration range, in order to prevent serious life-threatening infections.There are also shortcomings in this study, the patient’s child follow-up leads to the fact that his long-term follow-up results are not yet available, and we will continue to follow up to observe the changes in his tumor and also the long-term side effects.

## Author contributions

**Conceptualization:** Jun Cheng, Yun Zou.

**Data curation:** Jun Cheng, Yun Zou, Ronghua Fu, Pingliang Jin, Hanxiang Bai.

**Formal analysis:** Jun Cheng, Yun Zou.

**Methodology:** Hua Yuan.

**Resources:** Yun Zou, Ronghua Fu, Xiangqun Huang, Hua Yuan.

**Software:** Jun Cheng, Pingliang Jin, Mengyu Huang, Zhiping Wu, Hanxiang Bai.

**Supervision:** Hua Yuan.

**Visualization:** Jun Cheng, Mengyu Huang, Zhiping Wu.

**Writing – original draft:** Jun Cheng.

**Writing – review & editing:** Hua Yuan.
